# Variable Radiological Presentations of Acute Appendicitis With Epiploic Appendagitis as a Differential Diagnosis: A Case Series

**DOI:** 10.7759/cureus.85381

**Published:** 2025-06-04

**Authors:** Nishant Agarwal, Bodhisatwa Choudhuri, Nilanjana Datta Chaudhuri, Nirmalya Bagchi

**Affiliations:** 1 Emergency Medicine, Charnock Hospital, Kolkata, IND; 2 Critical Care and Rheumatology, Parkview Super Speciality Hospital, Kolkata, IND; 3 Radiology, Charnock Hospital, Kolkata, IND; 4 General Surgery, Charnock Hospital, Kolkata, IND

**Keywords:** abdominal pain, appendiceal diseases, appendicitis, appendicular abscess, differential diagnosis, epiploic appendagitis, fecal impaction, peritoneal diseases, radiology, tomography x-ray computed

## Abstract

Acute appendicitis (AA) is one of the most common causes of lower abdominal pain and requires prompt and accurate diagnosis to prevent complications. However, its clinical presentation and radiologic findings can vary widely, and the condition may be mimicked by other intra-abdominal pathologies. This case series illustrates the diverse presentations of acute appendicitis, emphasizing the variability in imaging findings, and highlights epiploic appendagitis as a rare but important differential diagnosis. The series underscores the critical role of advanced imaging, particularly contrast-enhanced CT, in ensuring accurate diagnosis and appropriate management.

## Introduction

Acute appendicitis is still one of the most prevalent causes of acute abdominal pain and the major reason for emergency surgery around the world [1,2]. Patients typically describe pain in the right lower quadrant (RLQ), as well as fever, nausea, and tenderness around McBurney's point. However, the clinical picture isn't always clear. Atypical appearances, particularly in older patients or those with atypical anatomy, such as situs inversus or intestinal malrotation, can complicate the diagnosis [3].

Ultrasound and contrast-enhanced CT (CECT) are the most commonly employed imaging techniques for determining a diagnosis [4]. While a large, inflamed appendix with surrounding fat stranding is the most common imaging observation, the radiologic appearance is not always consistent. Many intra-abdominal disorders can mimic appendicitis, both in terms of symptoms and scan results [3].

Primary epiploic appendagitis is an uncommon type of mimic. This benign condition develops when small fatty pouches lining the colon become inflamed due to torsion or venous obstruction [5-7]. Clinically, it can be mistaken for appendicitis since it involves RLQ pain, nausea, and a modest increase in white cell count [8,9]. However, it typically resolves on its own, and CT imaging can help distinguish it from more dangerous surgical disorders [6,10-12].

This case series analyses the varied atypical presentations of appendicitis and underlines the importance of considering epiploic appendagitis in the differential diagnosis. Early recognition of such distinctions can help clinicians avoid unnecessary procedures and provide appropriate care.

## Case presentation

Case 1

A 28-year-old male patient with no previous health concerns presented with a two-to-three-day history of acute discomfort localised in the right lower abdomen, accompanied by nausea. The examination indicated that the patient was stable, and abdominal palpation revealed significant tenderness in McBurney’s area, along with the presence of rebound tenderness. An earlier abdominal ultrasound revealed no abnormalities. Due to the ongoing symptoms and sustained clinical apprehension regarding appendicitis, a CECT scan was recommended. Oral, intravenous, and rectal contrast agents were employed for improved visualisation.

**Figure 1:**
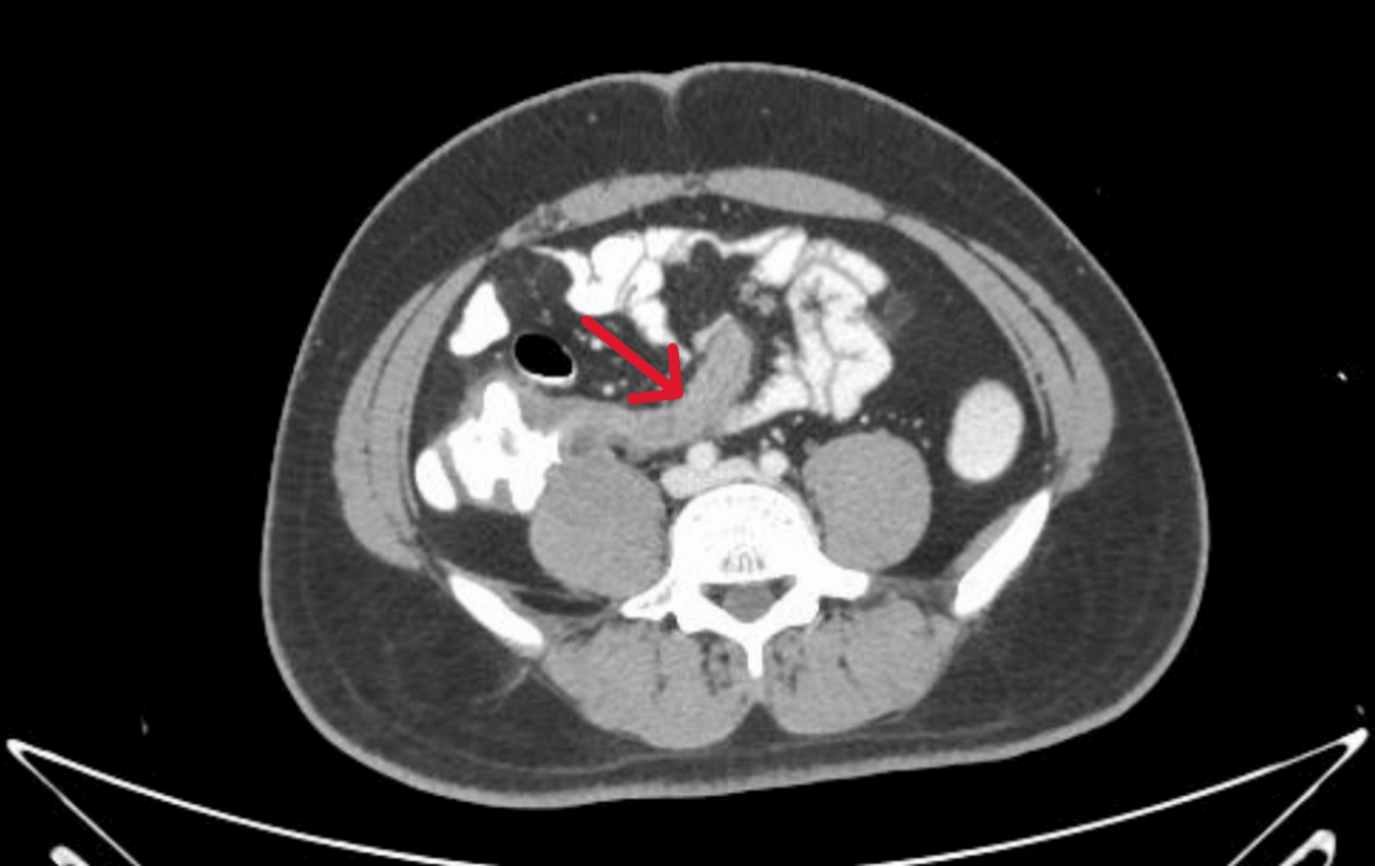
Contrast-Enhanced CT Depicting Acute Appendicitis with Periappendiceal Abscess (Case 1) Axial contrast-enhanced CT (CECT) of the abdomen shows a dilated and inflamed appendix (red arrow) with wall enhancement and surrounding fat stranding. A thick-walled hypodense collection adjacent to the appendix is consistent with a periappendiceal abscess. The caecal wall appears thickened, with associated mesenteric lymphadenopathy, indicating complicated appendicitis.

The CT scan (Figure 1) revealed a dilated, convoluted appendix exhibiting discernible wall enhancement. A robust periappendiceal abscess was detected at the appendix's base. Fat stranding was present in the surrounding tissue, with mural thickening affecting the neighbouring caecum and ileocecal junction, as well as increased mesenteric lymph nodes in the right iliac fossa. The results corroborated the diagnosis of acute appendicitis complicated by abscess development.

Despite the recommendation for additional therapy, the patient refused hospitalisation. He was discharged with antibiotics and comprehensive follow-up instructions. Three days later, he returned with alleviation of acute symptoms; nonetheless, a palpable mass was now observed in the right iliac fossa, indicative of an appendicular lump. A surgical consultation was obtained, and an interval appendicectomy was scheduled following conservative treatment.

Case 2

A 33-year-old previously healthy lady presented to the outpatient clinic with complaints of pain in her right lower abdomen that had persisted for two days. She also reported a low-grade fever and nausea. Her menstrual cycles were regular, with no signs of pregnancy. Physical examination revealed her to be haemodynamically stable, with a localised discomfort in the right iliac fossa.

**Figure 2:**
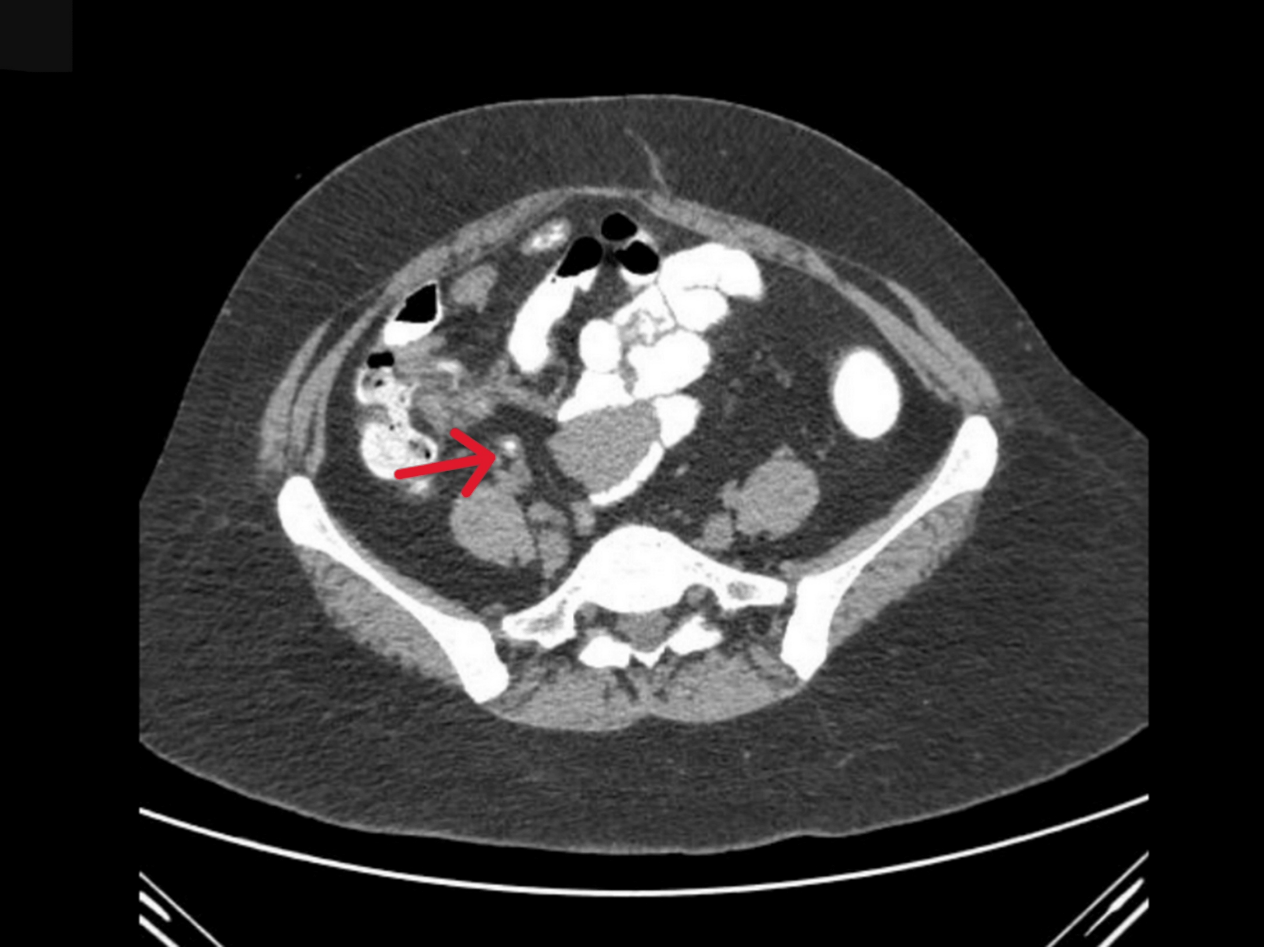
Contrast-Enhanced CT Showing an Appendicolith in Acute Appendicitis (Case 2) Axial CECT image reveals a distended appendix containing a hyperdense intraluminal appendicolith (red arrow), with periappendiceal fat stranding and small reactive mesenteric lymph nodes. These findings are typical of acute appendicitis with luminal obstruction due to a fecalith.

We used oral, intravenous, and rectal contrast to conduct a CECT scan of the abdomen for further investigation. The scan (Figure 2) revealed intraluminal faecaliths in the appendix and a few small, reactive mesenteric lymph nodes in the right iliac fossa. These data were interpreted as being compatible with acute appendicitis, most likely caused by faecolith blockage.

She was put on conservative treatment, which included intravenous antibiotics and pain relievers. We sought a surgical examination and, given her clinical stability, advised her to undergo an elective appendicectomy once the acute phase had passed.

Case 3

A 32-year-old woman with no prior medical history came to the outpatient department complaining of lower abdominal pain starting the day before. Although she reported not having a fever, she experienced nausea. Her menstrual cycles were regular, and no recent evidence pointed to pregnancy. Her vital signs were stable, and we noted tenderness in the right iliac fossa near McBurney's point. Although Rovsing's sign was negative, and there was no rebound tenderness.

**Figure 3:**
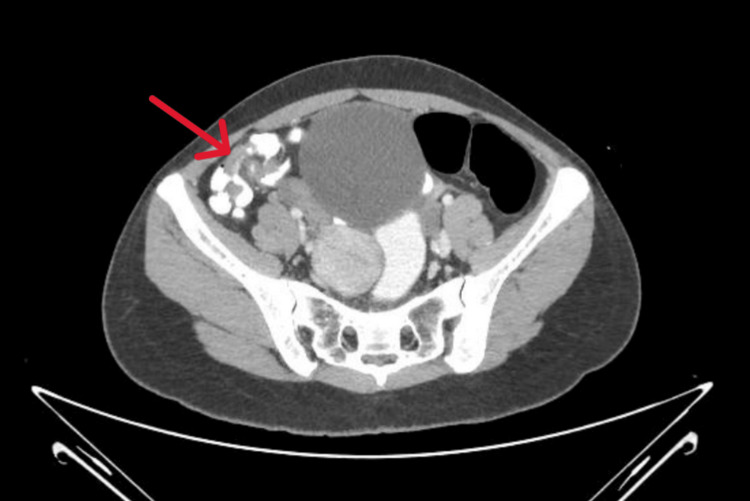
Contrast-Enhanced CT Demonstrating Mild Uncomplicated Appendicitis (Case 3) Axial CECT scan shows a mildly dilated appendix (red arrow) with heterogeneous wall enhancement and minimal periappendiceal fat stranding. No appendicolith or abscess is seen, consistent with early uncomplicated appendicitis.

Routine laboratory analyses, including serum beta-hCG, C-reactive protein, and complete blood count, were within normal limits. The first abdominal ultrasound was inconclusive since the appendix could not be seen. The right iliac fossa showed discomfort upon probing. The ambiguous ultrasound findings and persistent symptoms prompted a CECT scan of the abdomen using oral, intravenous, and rectal contrast agents. Findings suggestive of mild, uncomplicated appendicitis from the scan (Figure 3) showed a dilated, twisted appendix with slight heterogeneous wall enhancement, minor fat stranding in the periappendiceal region, and a few small mesenteric lymph nodes in the right iliac fossa.

Although surgical therapy was available, the patient opted for a non-operative approach. We started her on a conservative treatment plan using painkillers and antibiotics after a long discussion about the possible risks and benefits.

Case 4

**Figure 4:**
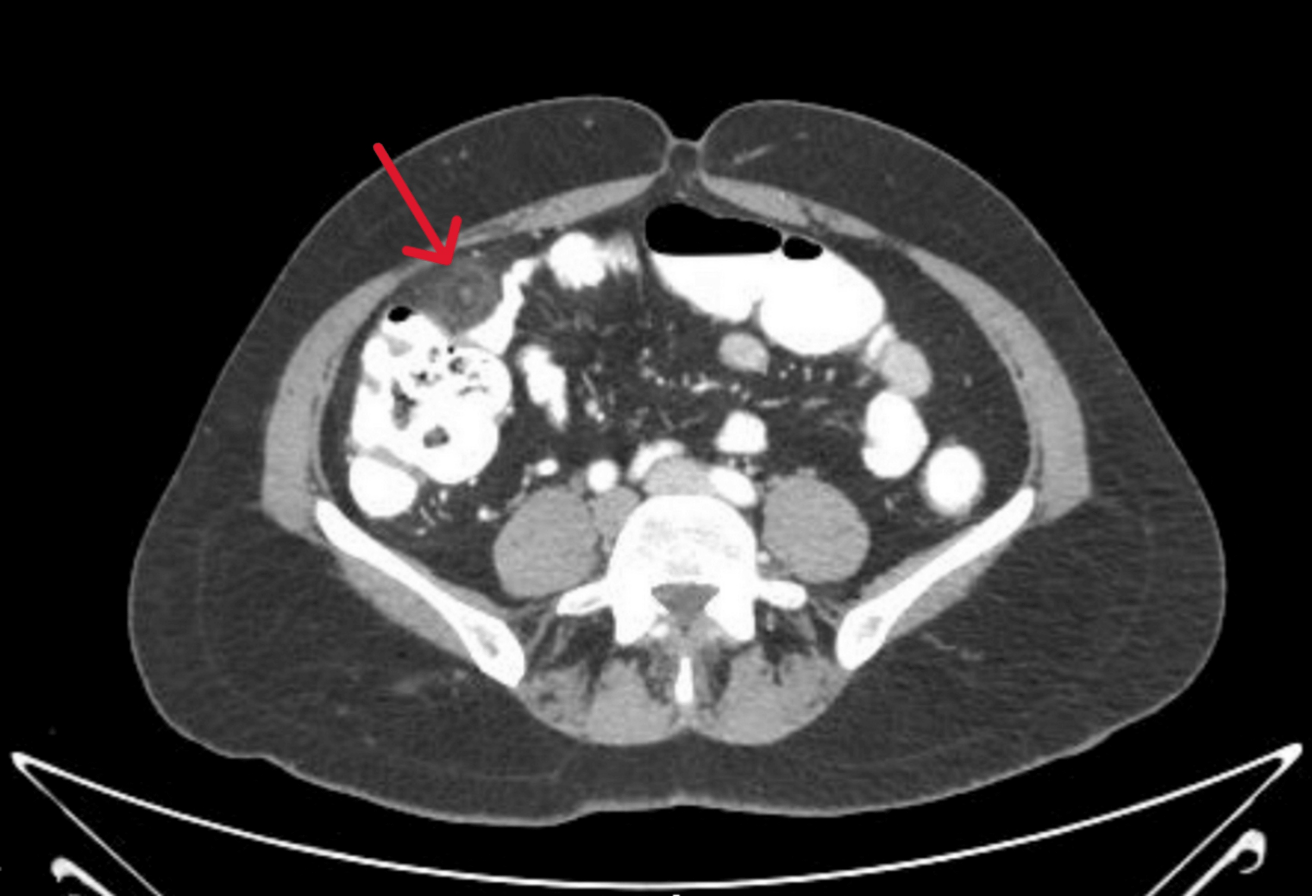
Contrast-Enhanced CT Revealing Epiploic Appendagitis Mimicking Acute Appendicitis (Case 4) Axial CECT image demonstrates a well-defined, oval, fat-attenuating lesion with a thin hyperattenuating rim (red arrow) adjacent to the ascending colon. The appendix appears normal in a retrocecal location. These findings are characteristic of epiploic appendagitis* *— a benign mimic of acute appendicitis.

A 53-year-old woman, previously in excellent health and without known chronic conditions, appeared with a sudden onset of severe lower abdomen pain that began the previous night. She experienced nausea and one episode of vomiting in addition to the discomfort. She did not report any pyrexia, urological issues, atypical vaginal haemorrhage, or gastrointestinal concerns. During the physical assessment, she exhibited haemodynamic stability. The right iliac fossa showed tenderness, but rebound tenderness and Rovsing's sign were not present. We conducted a CECT scan of the abdomen, utilising oral, intravenous, and rectal contrast for further examination. The scan (Figure 4) revealed an ill-defined, oval lesion of fat density with a hyperdense margin located anterior to the proximal ascending colon. The lesion induced indentation and slight mural thickening of the neighbouring colonic wall. We observed the appendix in a retrocecal position and found it completely normal, showing no signs of irritation. These results were indicative of epiploic appendagitis.

The patient had conservative treatment with intravenous antibiotics and painkillers. Her symptoms resolved rapidly, and she achieved complete recovery without surgical intervention.

## Discussion

Case 1 illustrates a severe manifestation of acute appendicitis, exacerbated by a periappendiceal abscess, presumably resulting from a delayed diagnosis and treatment. The preliminary abdominal ultrasound could not identify the pathology--an occurrence that is not unusual, particularly in patients with excessive intestinal gas or increased body mass, which can hinder visualisation of the appendix [1]. Notwithstanding this, clinical suspicion persisted, necessitating additional inquiry. The CECT scan was essential, demonstrating characteristic indicators of appendicitis, such as a dilated, inflamed appendix with wall enhancement, fat stranding, and a localised abscess [4]. The patient refused urgent admission, prompting the initiation of intravenous antibiotic therapy. The emergence of a discernible tumour in the right iliac fossa necessitated a surgical referral for interval appendicectomy. This example underscores the necessity of maintaining a high level of clinical attention and illustrates how prompt imaging and meticulous monitoring can influence outcomes in complex appendicitis.

Case 2 exemplifies a straightforward manifestation of appendicitis, linked to the presence of a faecalith - a known aetiology of luminal blockage in the appendix. Fecaliths, consisting of calcified and dehydrated stool, are commonly detected on CT scans and markedly elevate the probability of appendiceal inflammation when associated with right lower quadrant pain and mesenteric lymphadenopathy [1,4]. The diagnosis was clear, corroborated by clinical findings and imaging. Although surgery is the definitive therapy, the patient chose cautious maintenance with antibiotics, a decision increasingly accepted in certain stable instances [13,14]. Nonetheless, the existence of a faecalith necessitated vigilance due to the elevated risk of recurrence linked to non-surgical methods. We conducted a surgical consultation and recommended an elective appendectomy after clinical stabilisation.

Case 3 exemplifies a quintessential instance of simple acute appendicitis. Despite initial laboratory values, including leukocyte count and CRP, being within normal ranges, the patient had localised tenderness in McBurney's area and ongoing complaints. The observations, along with an unvisualised appendix on ultrasonography, warranted further imaging. The CT scan subsequently verified mild appendicitis with slight periappendiceal stranding and reactive mesenteric lymphadenopathy [3,4]. Despite the proposal for surgery, the patient chose a conservative approach and responded positively to antibiotics. This example emphasises the increasing endorsement of non-surgical treatment for uncomplicated appendicitis, particularly when clinical and imaging results are consistent, and illustrates the essential function of CT in instances where preliminary evaluations are ambiguous [1,13,14].

Case 4 presented a diagnostic problem since the patient's symptoms closely mirrored those of appendicitis, which included acute right iliac fossa pain, nausea, and localised tenderness. Based on this presentation, appendicitis was the primary clinical consideration. Nonetheless, cross-sectional imaging was pivotal. The CT scan did not corroborate the diagnosis of appendicitis; rather, it identified a fat-density, oval-shaped lesion encircled by a hyperdense rim--characteristics indicative of epiploic appendagitis [6,9]. We observed the appendix in a retrocecal location, and it appeared entirely normal. Timely identification of this self-limiting condition allowed for careful treatment without unnecessary surgery. This example underscores the necessity of evaluating appendicitis mimics, especially in middle-aged individuals, and illustrates how CT imaging can assist in distinguishing between surgical emergencies and benign conditions such as epiploic appendagitis [6,7].

Epiploic appendagitis, an unusual but benign and self-resolving inflammatory condition, is usually caused by torsion or spontaneous thrombosis of the epiploic appendages - small, fat-filled pouches of serosa lining the colon [5-7]. The most dense distribution of these appendages is near the recto-sigmoid junction and the ileocecal region [6]. Slightly more common in women and those with obesity, the condition typically affects adults aged 20-50 years, possibly due to the presence of more prominent or elongated appendages [5,7]. Torsion or venous thrombosis causes ischaemia, which causes localised fat necrosis and inflammation [6,7]. Clinical suspicion may be raised in patients presenting with sudden-onset, localised, non-migratory abdominal pain--most often in the left or right lower quadrant--without associated systemic signs, such as fever or leukocytosis. A history of similar self-limiting episodes, minimal gastrointestinal symptoms, and absence of prior abdominal surgery can further support consideration of epiploic appendagitis in the differential diagnosis. Depending on the location of the affected appendage, epiploic appendagitis can clinically closely resemble other causes for acute abdominal pain, including appendicitis or diverticulitis [9,15]. Patients usually present with discomfort in the right iliac fossa in right-sided instances, like those seen in this series, making it impossible to differentiate from acute appendicitis based only on symptoms [9,11]. Epiploic appendagitis might be underdiagnosed even if its estimated incidence is low - about 8.8 cases per million yearly [5,7]. It is thought to be responsible for 1-2% of all acute abdominal pain [7]. CT scans typically show it as a relatively tiny (1.5-3.5 cm), well-defined fat-density lesion surrounded by a hyperattenuating rim [6,9]. You may also see a central high-density focus, known as the "central dot sign," which indicates a thrombosed draining vein [9]. Occasionally, mild thickening or indentation of the adjacent colonic wall can be observed; usually, however, there is no sign of full-thickness bowel wall involvement [9]. Importantly, the sight of a normal appendix on imaging is a major indicator that helps eliminate appendicitis and other imitators [11,12]. Usually conservative in nature, management of this condition calls for simple analgesics or non-steroidal anti-inflammatory drugs (NSAIDs). Usually, antibiotics are not needed unless there is proof of secondary infection [9,11]. A misdiagnosis could result in needless procedures like hospital admission, antibiotic use, or perhaps surgery [7,11]. Reducing the incidence of negative appendectomies and unnecessary laparoscopic explorations can be greatly aided by increased awareness of this condition, especially among emergency doctors and radiologists, alongside timely and suitable imaging [6,10]. Table 1 summarises the main radiologic differentials for right iliac fossa pain.

**Table 1:** Radiological Features of Common Causes of Right Iliac Fossa Pain This table summarises characteristic imaging findings using contrast-enhanced CT and ultrasound for various differential diagnoses of right iliac fossa pain. It highlights key radiologic indicators used to distinguish acute appendicitis from other mimics [1,3-7,9,11,12,15]. Abbreviations: CECT, contrast-enhanced computed tomography; MRI, magnetic resonance imaging; USG, ultrasonography; RLQ, right lower quadrant; RIF, right iliac fossa; VUJ, vesicoureteric junction; *C. difficile*, *Clostridioides difficile*; beta-hCG, beta human chorionic gonadotropin; Tc-99m, technetium-99m; GI, gastrointestinal.

Condition	Primary Imaging Modality	Characteristic Radiological Features	Typical Patient Demographics / Clinical Clues
Acute appendicitis	CECT / ultrasound	Dilated appendix (>6 mm), wall thickening and enhancement, periappendiceal fat stranding, appendicolith, caecal tip thickening, mesenteric lymphadenopathy	Adolescents and young adults; pain starts periumbilically, localizes to RIF; anorexia, fever common
Crohn’s disease	CECT / MRI	Thickened terminal ileum with luminal narrowing, mucosal hyperenhancement, fibrofatty proliferation, skip lesions, RLQ lymphadenopathy	Young adults with chronic diarrhoea and weight loss; may have perianal disease
Mesenteric adenitis / enteritis	CECT	Thickened distal ileum, mucosal hyperenhancement, submucosal oedema, clustered mildly enlarged mesenteric nodes, normal appendix	Common in children; often follows viral infection or gastroenteritis
Diverticulitis (cecal/sigmoid)	CECT	Diverticula with pericolic fat stranding, bowel wall thickening, no submucosal oedema	More common in elderly; sigmoid > caecal; often recurrent episodes
Infectious colitis	CECT	Diffuse or segmental bowel wall thickening, low attenuation, pericolonic fat stranding	Recent antibiotic use or gastroenteritis; C. difficile in hospitalized patients
Epiploic Appendagitis	CECT	Oval fat-density lesion with hyperattenuating rim and central dot sign, adjacent colonic indentation, normal appendix	Middle-aged, often overweight; sudden focal pain without systemic symptoms
Omental infarction	CECT	Larger fat-density mass than epiploic appendagitis, enhancing capsule, minimal bowel involvement	Right sided or central pain; mimics appendicitis
Urolithiasis	Non-contrast CT / ultrasound	Ureteric or VUJ stone, proximal ureter dilation, perinephric fat stranding, hydronephrosis	Sudden colicky flank pain radiating to groin; haematuria common
Ovarian cyst / torsion	Transvaginal USG / CECT	Cystic adnexal lesion with thickened wall, echogenic content or haemorrhage, decreased ovarian perfusion	Reproductive-age females; sudden severe pelvic pain; may follow exertion
Ruptured ectopic pregnancy	Transvaginal ultrasound	Adnexal mass, free fluid in pelvis, no intrauterine gestation, positive beta-hCG	Missed period, reproductive-age female; may present in shock
Endometriosis	Ultrasound / MRI	Heterogeneous adnexal mass, solid-cystic components, pelvic inflammation	Cyclical pelvic pain linked to menstruation; may have infertility
Pelvic inflammatory disease	Transvaginal USG / CT	Fluid-filled, dilated fallopian tubes, tubo-ovarian abscess, loss of fat planes	Young sexually active women; fever, cervical motion tenderness
Meckel’s Diverticulitis	CECT / Scintigraphy	Blind-ending fluid-filled ileal structure, wall thickening, fat stranding; Tc-99m shows ectopic gastric mucosa	Paediatric or young adults; painless GI bleeding or RIF pain
Yersinia Enterocolitica Infection	CECT	Terminal ileitis, RLQ lymphadenopathy, normal appendix	Mimics Crohn’s; recent pork ingestion or travel
Mittelschmerz (Ovulatory Pain)	Transvaginal Ultrasound	Normal ovary ± small follicular cyst; minimal free fluid	Mid-cycle pain in young women; self-limiting
Abdominal Wall Hematoma	Ultrasound / CECT	Heterogeneous mass in abdominal wall (e.g., rectus sheath); no intra-abdominal involvement	Trauma or anticoagulation; localized tenderness, possible ecchymosis
Psoas Abscess	CECT	Rim-enhancing collection in or around psoas, may extend to iliacus or hip joint	Fever, flank/hip pain, difficulty walking; positive psoas sign

This case series shows the various ways acute appendicitis can appear in imaging and points out that epiploic appendagitis is an important but often overlooked differential diagnosis. It emphasises the need for a CECT scan to precisely determine the source of abdominal pain and recommend suitable therapy [4]. In chosen situations, correct imaging diagnosis might prevent surgery and assist conservative management of non-surgical conditions.

## Conclusions

From textbook clinical characteristics to more subtle or complex presentations, acute appendicitis may manifest in many ways. Although imaging techniques - especially ultrasound and CECT - are crucial for diagnosis, they are not perfect. Sometimes, particularly in the early phases or with unusual anatomy, imaging might not offer a conclusive response. Rarer diseases like epiploic appendagitis, which can closely mimic appendicitis in both symptoms and location of discomfort, add to the complexity.

Maintaining a high degree of clinical suspicion is essential in such cases. In the framework of clinical findings, careful interpretation of imaging can significantly raise diagnostic accuracy. Differentiating true appendicitis from its mimics at an early stage lets doctors customise management correctly, ensuring timely surgical intervention when necessary, while avoiding it in cases better suited for conservative care. In the end, this strategy lowers the possibility of pointless surgeries and promotes better results.
